# Ultrasound-Guided Fascia Iliaca Compartment Block Simulation Training in an Emergency Medicine Residency Program

**DOI:** 10.7759/cureus.52411

**Published:** 2024-01-16

**Authors:** Bumin Kong, Sophia Zabadayev, Joshua Perese, Ajit Panag, Zan Jafry

**Affiliations:** 1 Emergency Department, Loma Linda University Medical Center, Loma Linda, USA

**Keywords:** ultrasound-guided, peripheral nerve block, simulation medicine, emergency medicine physician, facia iliaca compartment block

## Abstract

Introduction

Geriatric hip fractures present a difficult challenge in the emergency department (ED) to achieve adequate analgesia. Opioid-sparing ultrasound-guided fascia iliaca compartment blocks (UFIB) have been shown to be both safe and effective in treating pain from hip fractures. In this study, we investigated the teachability of UFIB to emergency medicine (EM) residents using simulation models and also assessed if UFIB training increases its utility in the ED.

Methods

We created a UFIB model to simulate the procedure in a controlled environment. Sixteen residents from Loma Linda Emergency Medicine Residency participated in a pre-workshop survey and hands-on UFIB workshop. Comfort level in performing UFIB and confidence level in needle finding skills during UFIB were analyzed, plotted, and represented graphically.

Results

Comfort level in performing UFIB increased by approximately 50% (p < 0.01). Success rates also increased by 460% (p<0.05) after the workshop. However, the UFIB continued to be underutilized as 44% of respondents expressed that there is a “lack of time” to perform UFIB during their shifts.

Conclusion

A single one-hour workshop increased comfort level in performing UFIB and helped residents successfully achieve better pain control in patients with hip fractures. However, residents continued to refrain from using UFIB because it is too time-consuming.

## Introduction

Geriatric hip fractures are common injuries, causing significant pain that is often inadequately treated [1,2]. The mainstay of treatment is to use opioids in conjunction with non-steroidal anti-inflammatory drugs (NSAIDs), acetaminophen, and regional anesthesia. Opioids, acetaminophen, or NSAIDs are often inadequate or have significant side-effects including respiratory depression, altered mental status, hypotension, and increased risk of bleeding [3]. By contrast, fascia iliaca compartment block has been shown to be a safe and efficacious opioid-sparing modality that provides superior analgesia compared to systemic opioids [4-6]. A recent study demonstrated that fascia iliaca compartment blocks using the loss of resistance technique were easy to teach junior residents and led to a 75% success rate in achieving greater than 50% analgesia [7]. The use of ultrasound further increased the safety profile of nerve blocks by not only increasing the first-pass success rate but also decreased the amount of local anesthetic needed to achieve analgesia [8-10]. Recent studies also showed that medical training on simulation models is an effective modality to learn ultrasound-guided procedures such as central venous catheter insertion, soft tissue abscess drainage, and foreign body removal [11,12]. In fact, a study showed that ultrasound-guided fascia iliaca compartment blocks (UFIB) can be taught to residents and medical students using a simulation model [13]. However, no research has been done to assess if this education module affected how readily UFIB is performed in clinical practice. Our research attempts to reevaluate the efficacy of teaching UFIB to EM residents through the use of simulation models but also assess if such training affects the frequency of UFIB being performed in the ED.

## Materials and methods

Study design

All protocols were reviewed and approved by the Loma Linda University Health Institutional Review Board (IRB#5220198). Survey questions were developed in conjunction with faculty members of the Advanced Emergency Medicine Ultrasound Fellowship. Questions were aimed at assessing the comfort level of UFIB, experience with peripheral nerve blocks, and perceived barriers to performing UFIB in the ED.

Simulation model details

**Figure 1:**
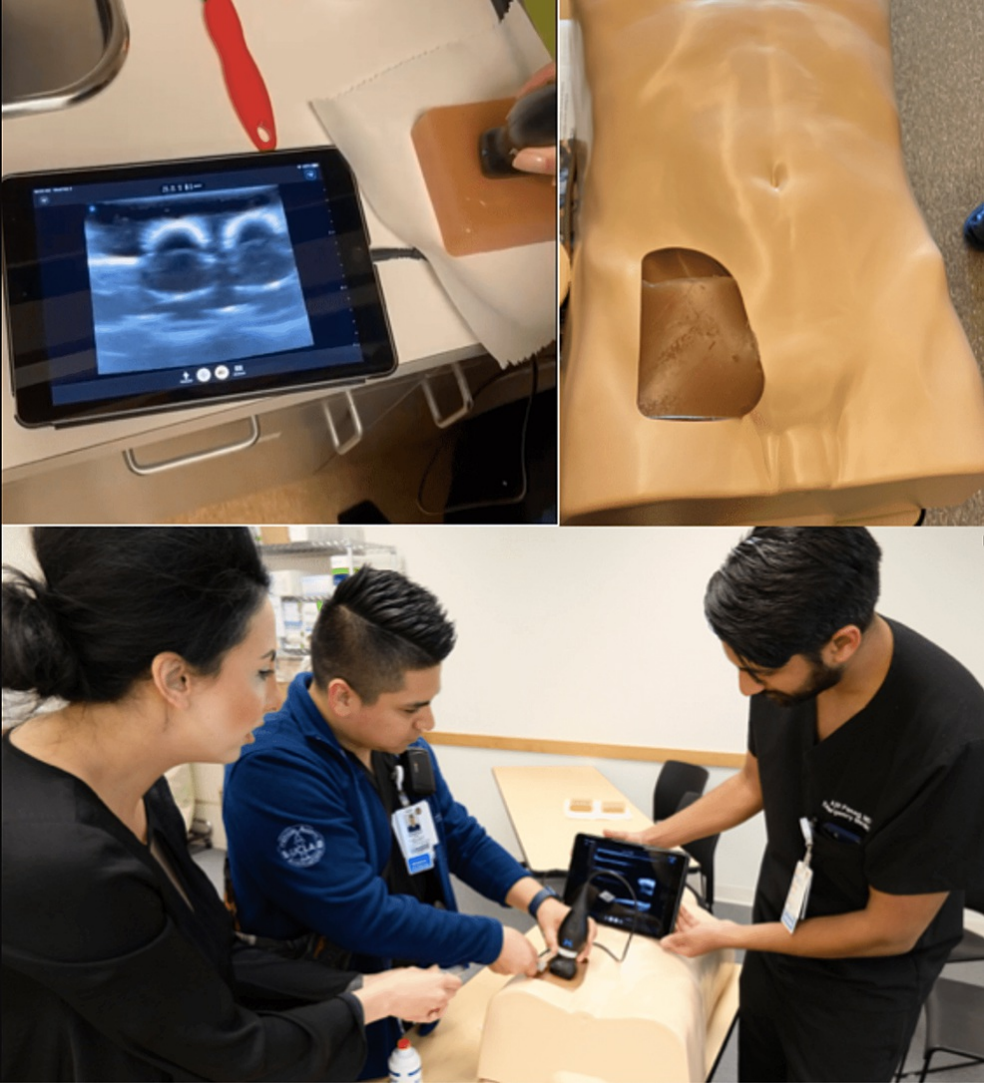
Simulation model placed in a human trainer model to practice Ultrasound-Guided Fascia Iliaca Compartment Blocks

**Figure 2:**
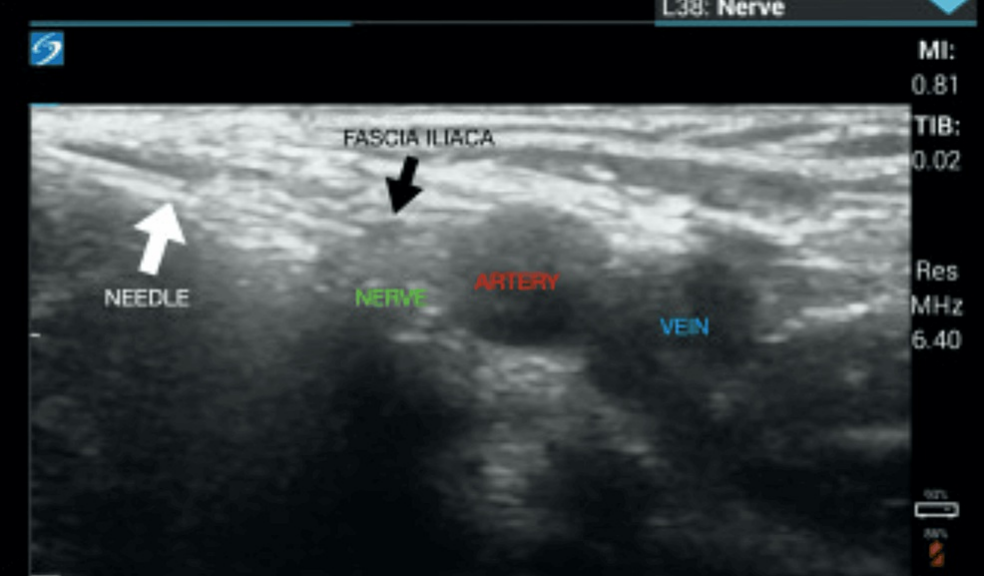
Ultrasound image of a patient's fascia iliaca compartment

Simulation models were made in conjunction with an affiliated medical school simulation center. Ballistic and silicone gelatins were melted using a slow cooker. Melted ballistic gelatin was poured over a square mold and melted silicone gelatin was poured into two 3cc syringes to form the femoral artery and the femoral vein. Another 3cc syringe was filled with melted silicone gelatin infused with corn starch to make the femoral nerve. Once settled, the femoral nerve was placed over the molded ballistic gelatin and then a gauze was placed on top to mimic the fascia iliaca. The femoral artery and vein were placed over the fascia iliaca gauze, medial to the femoral nerve. More melted ballistic gelatin was poured over the femoral vessels and then another gauze was placed over the model to mimic the fascia lata. More melted ballistic gelatin was poured over the fascia lata gauze to complete the simulation model. Several simulation models were built using parchment paper instead of gauze, a method that was also well-received by residents. Lastly, the simulation models were placed in a human trainer model that was initially created to practice placing femoral central venous catheters (Figure 1). Ultrasound image of an actual patient is also added for comparison (Figure 2).

Study protocol

The UFIB workshop took place at a simulation center during a weekly education conference. Sixteen EM residents, varying from postgraduate level 1 to 3, agreed to participate in this study. Prior to performing UFIB on simulation models, every resident completed a pre-workshop survey. Residents were then given a presentation regarding the nerve block’s indications, contraindications, risks and benefits, relative anatomy, and the actual steps of performing UFIB. Residents were given approximately one hour to practice UFIB on simulation models. During subsequent clinical shifts, residents were able to perform UFIB with their respective EM attending physician. Two months after the workshop, residents completed a post-workshop survey. EM-attending physicians were also given an option to participate in additional training in UFIB in order to better supervise and encourage residents to perform UFIB during shifts.

Data analysis

The pre-workshop survey asked numerous questions including the residents’ comfort level and experience in performing UFIB, proficiency in needle-finding skills for an in-plane approach during UFIB, likelihood of performing UFIB on appropriate patients, and barriers to performing UFIB on appropriate patients. The post-workshop survey asked these questions but also asked to recall the residents’ success rates in UFIB before and after the workshop training. Answers were converted to a quantifiable measure by using Likert scales. Two-tailed Mann-Whitney U tests were performed to determine the statistical significance between pre/post-workshop means. Statistical significance was defined as p-value < 0.05. Three open-ended questions in the survey offered participants an opportunity to write and further elaborate on their responses.

## Results

**Figure 3:**
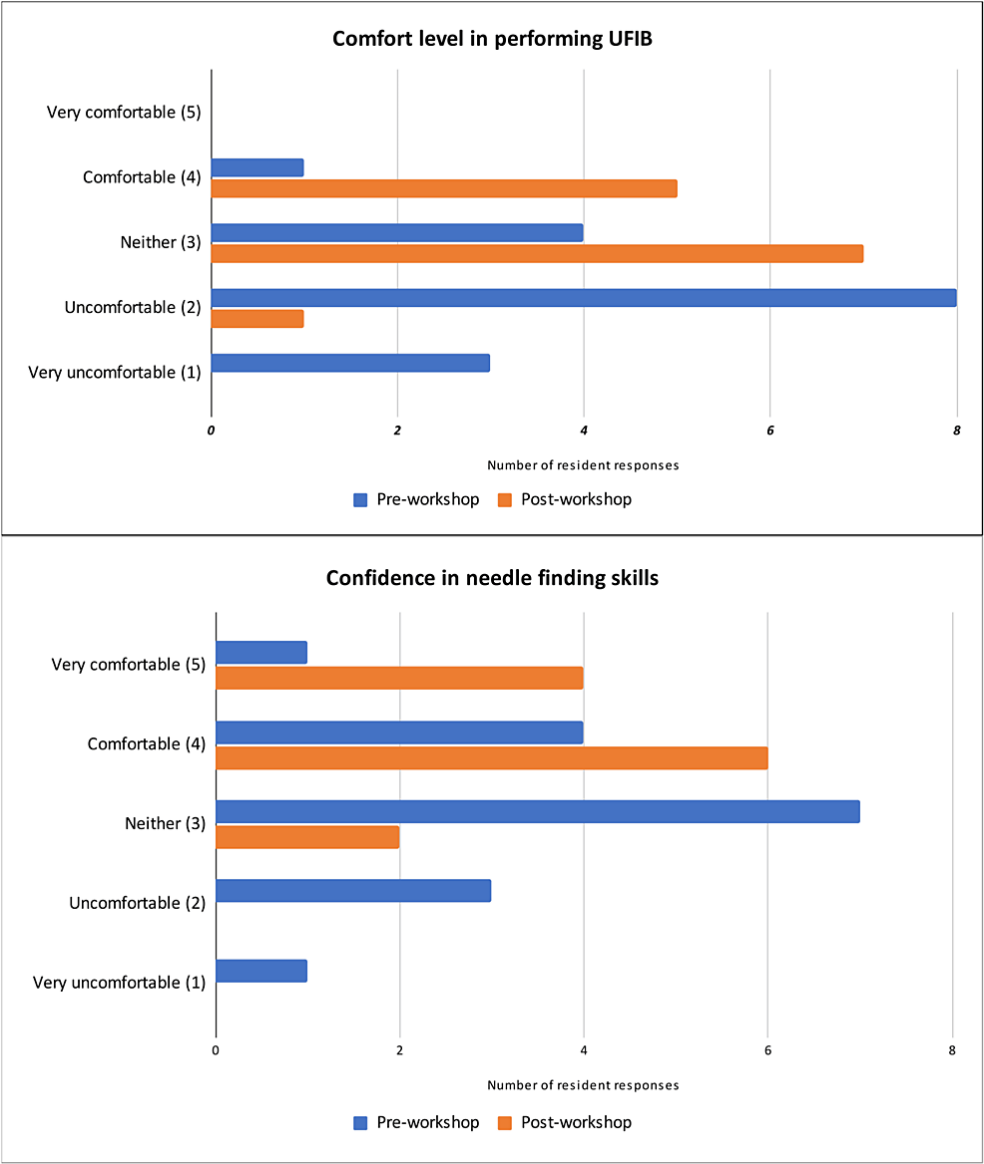
Comfort level in performing Ultrasound-Guided Fascia Iliaca Compartment Block (UFIB) and confidence level in needle finding skills for in-plane technique. The median comfort levels pre- and post-workshop were 2 and 3, respectively. The average comfort levels pre- and post-workshop were 2.19 and 3.31, respectively; the distributions in the two groups differed significantly (Mann-Whitney U=12, N1=16, N2=13, p<0.05). The median confidence levels for needle finding skills pre- and post-workshop were 3 and 4, respectively; the distributions in the two groups differed significantly (Mann-Whitney U=34, N1=16, N2=10, p<0.05).

Sixteen residents completed the pre-workshop survey and the UFIB workshop while 13 of the 16 residents participated in the post-workshop survey, for a completion rate of 81.25%. Median comfort levels in the pre-workshop group and the post-workshop group were 2.2 and 3.3, respectively; the distributions in the two groups differed significantly (Mann-Whitney U=12, N_1_=16, N_2_=13, p<0.05). Median confidence level in residents’ needle-finding skills during the in-plane approach for UFIB before and after the workshop were 3.1 and 4.2, respectively; the distributions in the two groups differed significantly (Mann-Whitney U=34, N_1_=16, N_2_=10, p<0.05) (Figure 3). Residents were asked to recall and approximate their success rates of UFIB before and after the workshop. Success was defined as achieving 50% or more of analgesia according to their patients. In our pre-workshop surveys, four residents self-reported that their success rates ranged from 25-33%. In our post-workshop survey, four residents self-reported that their success rates ranged from 50-100%.

**Figure 4:**
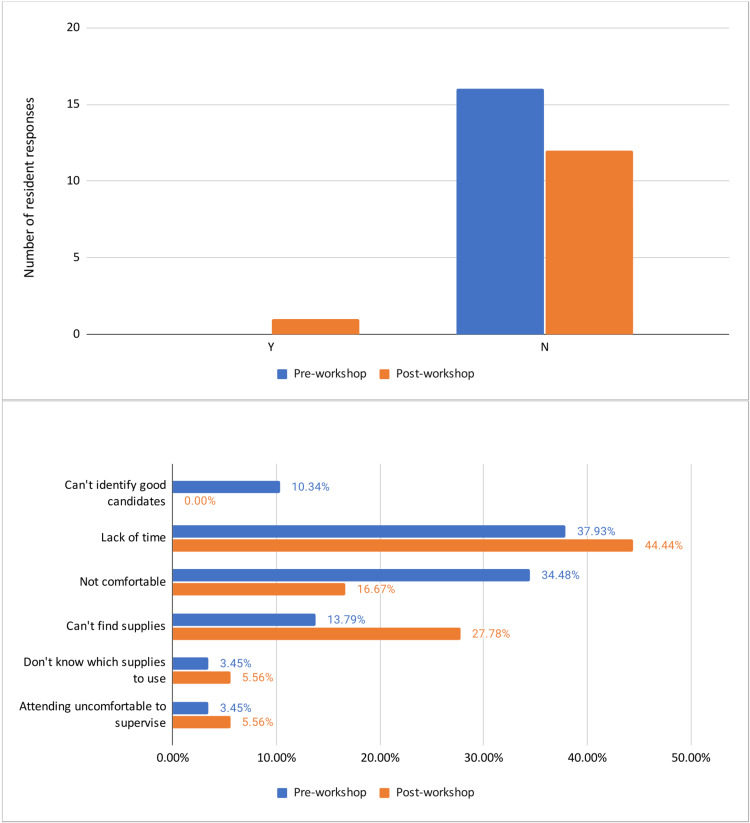
Is Ultrasound-Guided Fascia Iliaca Compartment Block (UFIB) routinely performed on all patients who are good candidates? If not, why? Pre- and post-workshop averages were 0.00 and 0.125, respectively, with a p-value of 0.077. “Lack of time” remained the most common barrier while the second most common barrier changed from “Not comfortable” to “Can’t find supplies” after the workshop.

Interestingly, UFIB was not routinely performed on all patients who are strong candidates for the nerve block (Figure 4). When inquired about the possible barriers faced in performing UFIB prior to the workshop, “lack of time” was the most common response (37.9%), followed closely by “not comfortable” (34.5%). After the workshop, “lack of time” continued to be the most common response at 44.4%, but the second most common response changed to “can’t find supplies” at 27.8% (Figure 4).

## Discussion

Two months following the UFIB workshop, residents reported an increase in their comfort level in performing UFIB by over 50% and confidence level in their needle-finding skills using the in-plane approach by over 35%. This increase in confidence in performing ultrasound-guided peripheral nerve blocks is consistent with a recently published study. Tucker et al. (2021) reported that a week-long ultrasound-guided regional anesthesia elective increased the confidence level of every trainee, who was later “likely” or “very likely” to implement ultrasound-guided nerve blocks into his/her own future practice [14]. Arguably, these workshops and electives can increase the utilization of peripheral nerve blocks and influence a paradigm shift in acute pain management in the ED.

Reported comfort level in performing peripheral nerve blocks seemed to directly correlate with the success rates of UFIB during shifts. Although asking about success rates of prior UFIB introduces recall bias, this result can still be extrapolated to reflect the increased aptitude of residents in their skills in UFIB. This finding is congruent with recent studies that have illustrated the efficacy of simulation model training in teaching medical procedures such as central venous catheter insertion, soft tissue abscess drainage, and foreign body removal [11,12]. Certainly, a future study is needed that documents every UFIB performed by residents before and after the teaching workshop and subsequently surveys the pain level of patients who have undergone UFIB.

Despite the increase in their skills and confidence levels, EM residents continue to refrain from performing UFIB routinely on all patients who are good candidates. The most common barrier in performing UFIB in the ED was the “lack of time” to perform this procedure. Several studies have also demonstrated that one of the leading barriers in performing fascia iliaca compartment blocks is the significant time it takes from preparation to actual procedure [15,16]. Unfortunately, this workshop did not seem to have an impact on how often UFIB is performed in the ED. This finding from an academic institution will probably be accentuated in single coverage hospitals where a single EM physician has to manage not only a potentially busy ED but also has to treat critically ill patients in the intensive care unit. Performing nerve blocks can potentially delay EM physicians from seeing more patients in a prompt fashion.

In addition to the time it takes to perform UFIB, this study also identified that the inability to find the correct supplies prevented EM residents from performing UFIB. Although institutional variabilities exist, most emergency departments do not have echogenic nerve block needles readily available for use. Instead, these specialized needles may be located in a central supply room, thus posing an additional barrier to performing the procedure. Furthermore, additional supplies required for the procedure may be located in different storage spaces creating more inefficiencies and delays.

A potential way to combat this issue is by making the supplies readily available to perform peripheral nerve blocks. A procedure cart with sterile skin preparation solution, ultrasound cover and gel, gloves and towels, echogenic needles, and syringes with IV tubing should be readily available. This idea comes from a recent study that showed that having a plastic surgery-dedicated procedural cart not only decreased the time it took for plastic surgeons to perform laceration repairs in the ED but also reduced infection rate and improved overall cosmesis [17]. The authors' institution recently has built a similar "nerve block box" that has all the supplies readily available.

The authors’ institution also recently started an ED elective for regional anesthesia, emulating elements of an anesthesiology acute pain fellowship. This new concept of having a dedicated EM physician readily available to perform peripheral nerve blocks using a "nerve block box" within reach will hopefully encourage peripheral nerve blocks in the ED and also enhance patients’ experience and satisfaction with their care. Attending EM physicians self-reported that more residents have been performing peripheral nerve blocks after these initiatives have been organized.

Limitations

The small sample size is a major limitation of this study. In addition, there is a selection bias because only residents from one hospital were included in this study. This EM residency is a three-year academic program and has a strong ultrasound presence. Residents have exposure to many core and advanced modalities including resuscitative TEE and VExUS. Faculty have developed prominent educational tools and pioneered many of these advanced ultrasound techniques. As such, our residents do not represent EM residents from non-academic programs, four-year residencies, or residencies without a strong ultrasound presence. A follow-up randomized, prospective, controlled study with a larger sample size that includes both three and four-year programs would be needed to confirm these preliminary results.

Another limitation of this study is the timing at which attendings were offered refresher training in UFIB. Pre-nerve block surveys and the nerve block workshop were both performed by residents on March 24th, 2022, with the expectation to complete the post-nerve block surveys on May 31st, 2022. This gave residents roughly two months to perform UFIBs during their shifts. However, for residents to practice UFIB during their shifts, attendings needed to be comfortable supervising the procedure. Therefore, EM attendings were also offered a refresher course in UFIB. Due to scheduling conflicts, attending training occurred on May 12th, 2022. If the attending workshop had occurred earlier, residents would have had a longer time period with attendings being more comfortable supervising UFIB. Residents could have potentially practiced UFIB more often in the ED which may have influenced the results of this study.

Lastly, there were only four responses in both pre-workshop survey and post-workshop survey with regard to success rates in performing UFIB. Because of the anonymous nature of the surveys, it is unclear if the four residents who responded to the pre-workshop survey were the same four residents who responded to the post-workshop survey. Therefore, a head-to-head comparison cannot be made. Also, residents were asked to recall their past success rates in performing UFIB which clearly would be subject to recall bias.

## Conclusions

Geriatric hip fractures are extremely painful injuries that are commonly encountered in the ED. UFIB is a safe procedure with high efficacy that also reduces the use of potentially harmful opioids in elderly patients. This study demonstrates that UFIB can be easily taught to residents using simulation models. A single one-hour workshop increased comfort level in performing UFIB and helped residents successfully achieve better pain control in patients with hip fractures. However, the workshop failed to increase the frequency of UFIB performed in the ED.

## Appendices

**Table 1:** UFIB research data

Comfort level performing UFIB	Pre-workshop	Post-workshop
Very uncomfortable (1)	3	0
Uncomfortable (2)	8	1
Neither (3)	4	7
Comfortable (4)	1	5
Very comfortable (5)	0	0

N1	16
N2	13
N1*N2	208
N1+1	17
N2+1	14
[N1*(N1+1)]/2	136
[N2*(N2+1)]/2	91
R1	148
R2	287
U1	196
U2	12
Ustat	12
Ucritical	59

How would you rate your needle-finding skills for the in-plane technique	Pre	Post
Very comfortable (5)	1	4
Comfortable (4)	4	6
Neither (3)	7	2
Uncomfortable (2)	3	0
Very uncomfortable (1)	1	0

N1	16
N2	10
N1*N2	160
N1+1	17
N2+1	11
Middle term 1	136
Middle term 2	55
R1	170
R2	181
U1	126
U2	34
Ustat	34
Ucritical	42

What is your success rate before and after workshop?				
Pre-workshop	33	25	25	33
Pre-workshop average	29			
Post-workshop	100	75	100	50
Post-workshop average	81.25			

Do you routinely perform UFIB on all pts who would be good candidates?	Y	N
Pre-workshop	0	16
Post-workshop	1	12

If not, what barriers do you have to perform UFIB?	Pre-workshop	Post-workshop
Can't identify good candidates	3	0
Lack of time	11	8
Not comfortable	10	3
Can't find supplies	4	5
Don't know which supplies to use	1	1
Attending uncomfortable to supervise	1	1
Total response	29	18
	percentage	percentage
Can't identify good candidates	10.34%	0.00%
Lack of time	37.93%	44.44%
Not comfortable	34.48%	16.67%
Can't find supplies	13.79%	27.78%
Don't know which supplies to use	3.45%	5.56%
Attending uncomfortable to supervise	3.45%	5.56%
